# Lifestyle indices of body composition and obesity risk and prevalence among multi-ethnic adolescents in Malaysia

**DOI:** 10.3389/fped.2022.899014

**Published:** 2022-11-01

**Authors:** Mohamed S. Zulfarina, Razinah Sharif, Sabarul Afian Mokhtar, Ahmad Nazrun Shuid, Isa Naina-Mohamed

**Affiliations:** ^1^ Pharmacoepidemiology and Drug Safety Unit, Department of Pharmacology, Faculty of Medicine, Universiti Kebangsaan Malaysia Medical Centre, Cheras, Malaysia; ^2^ Nutritional Science Program and Centre for Healthy Ageing and Wellness, Faculty of Health Sciences, Universiti Kebangsaan Malaysia, Kuala Lumpur, Malaysia; ^3^ Pro Vice-Chancellor’s Office, Health Campus, Cheras, Malaysia; ^4^ Department of Pharmacology, Faculty of Medicine, Universiti Teknologi MARA, Sungai Buloh, Malaysia

**Keywords:** obese, body fat, LEAN MASS, physical activity, sedentary behavior, antioxidants

## Abstract

The prevalence of childhood obesity in Malaysia has doubled in less than a decade. Moreover, being overweight and obese have been associated with immediate and later comorbidities, thus emphasizing the need to prevent obesity from an early age. This cross-sectional study involved 923 multi-ethnic Malaysian adolescents aged between 15 and 17 years old. Body composition was estimated using bioelectrical impedance analysis. Body mass index (BMI) classification was based on the World Health Organization (WHO) growth reference and the International Obesity Task Force (IOTF) cut-off. Meanwhile, the Child Growth Foundation (CGF) body fat reference was used to classify adolescents’ adiposity. Lifestyle indices including physical activity, sedentary behavior, supplement intake, smoking and alcohol status were assessed *via* questionnaires. A high prevalence of overweight/obesity among the adolescents was observed according to the CGF (26%), followed by that of the WHO (24%) and then the IOTF (23%) cut-off, with high concordance values between each pair. After adjustment, a significant association was found between physical activity (PAQ score) and lean mass (*p* = 0.027). No lifestyle determinant was found to be a significant predictor of fat percentage. A high sedentary level increased the likelihood of obesity (OR 3.0, *p* < 0.01), while antioxidant-rich supplements were found to protect against obesity (OR 0.4, *p* < 0.05). The lifestyle predictors identified in this study may be considered when designing interventions that integrate lifestyle modifications targeting adolescents.

## Introduction

Obesity refers to excessive fat accumulation that might threaten a person's health (1). The rate of obesity has doubled in more than 70 countries since 1980 and has increased continuously to reach pandemic levels (2). In 2016, over 650 million adults and 124 million children and adolescents aged 5–19 (6% of girls and 8% of boys) were obese (3). In just four decades, childhood obesity has surpassed adult obesity tenfold (2). Many low- and middle-income countries (LMICs) are now facing the dual-burden of malnutrition, since obesity and overweight rates are rising parallel to the existing burden of undernutrition (4). Included in the affected countries is Malaysia, where the prevalence of overweight and obesity has risen exponentially, with half of the population (50.1%) being classified as overweight and obese in 2019 (5). Alarmingly, the obesity prevalence among Malaysian children under 18 has doubled in less than a decade (6, 7), hitting a record high of 14.8% in 2019 (5).

For many countries, the steep upsurge in obesity has been attributed to rapid nutritional transition (8). The fundamental cause of obesity is a long-term energy imbalance between the calories consumed (increased energy intake) and calories expended (reduced energy expenditure) (9, 10). The overconsumption of calorie-dense food and beverages, accompanied by physical inactivity and sedentary lifestyle preferences that lead to obesogenic environments, plays a pivotal role in the prevalence of obesity (11). Hence positive lifestyle changes are likely to be major factors in the prevention of obesity.

Obesity trends in children and adolescents are of particular concern for the predictions of how the burden of obesity might soon affect the population (9). Overweight and obese children/adolescents are more likely to become obese adults (12) and are highly associated with the risk of developing other non-communicable diseases (NCDs) such as cardiovascular disease, type 2 diabetes, and dyslipidemia, which eventually contribute to mortality and premature death (9, 8, 11, 13). Obesity is the fifth leading cause of global mortality (14), of which over 80% of premature deaths before the age of 70 occur in LMICs (15). Accordingly, obesity is now classified as a chronic progressive disease, distinct from being a risk factor for other diseases (16). In that regard, the prevention of childhood obesity is one of the key strategies for early intervention for NCDs.

Several local studies have been conducted to examine the lifestyle predictors of obesity risk among school-going adolescents living in certain states of Malaysia. Lifestyle determinants including low physical activity and smoking status have been associated with greater odds of obesity (17, l8). In an earlier study, lower physical activity and amount of moderate to vigorous physical activity (MVPA) in males, and high sedentary behavior (measured as screen time) in females have been linked with higher odds of obesity in adolescents (19). The available findings, mainly conducted among adolescents in the sub-urban regions of Malaysia, are relatively insufficient and warrant further studies. In recent years, concerns have arisen about the changes in adolescents’ physical activity following the COVID-19 pandemic (20, 21). Thus, it is crucial to revisit the classical (physical activity) and emerging lifestyle predictors, such as sedentary behavior. This study aims to evaluate the lifestyle indices associated with obesity risk and body composition (body fat and lean mass) among multi-ethnic adolescents in Malaysia and to compare the difference between international references when classifying adolescents' weight status. Monitoring childhood obesity and understanding the factors that contribute to this global epidemic may help public health efforts to formulate policies and implement culturally appropriate programs (22).

## Materials and methods

### Study design and sampling

This population-based, cross-sectional study was conducted among 15–17 years old Malaysian adolescents attending their fourth year (Form 4) of government secondary school education. A stratified random sampling technique was used to select six secondary schools [daily school-urban, daily school-rural, boarding, daily-vernacular, sports school, and Orang Asli (indigenous population) dominated school] to represent the national school system, locations, and heterogeneity of the population using computer-generated random number lists. The adolescents were recruited from three regions: the Federal Territory of Kuala Lumpur (metropolitan area), Selangor (central region), and Pahang (eastern region), based on discussions with the Ministry of Education (MOE) of Malaysia (23, 24). All the fourth-year students in the selected secondary schools were eligible to participate and be included in the current study if they were healthy and had no chronic disease that could prevent them from being physically active. The subjects were informed of the study and their participation was voluntary. Written informed consent was obtained from the participants and their legal guardians before the inception of this study. In summary, of the 1,078 adolescents invited, 960 adolescents returned with a signed consent form and had a complete data set, while 37 were excluded because 22 adolescents were not in the age range and 15 were non-Malaysians/non-residents of Peninsular Malaysia. Hence, 923 adolescents were included in the final analysis.

The study was conducted in accordance with the Declaration of Helsinki and approved by the Institutional Ethics Committee of the University Kebangsaan Malaysia Medical Center (UKMMC) (UKM 1.5.3.5/244/FF-2014). Formal approvals were also obtained from the Ministry of Education (MOE), the Department of State Education (JPN), the Department of Orang Asli Development (JAKOA), and the school authorities.

### Anthropometric and body composition

The heights and weights of the participants were measured in triplicate by the same trained personnel in each respective school. Height was measured using a portable Stadiometer to the nearest 0.1 cm (Seca 213, Germany). Body weight was measured in light clothing to the nearest 0.1 kg using a segmental body composition analyzer BC-420 MA (Tanita Corporation, Japan). Age- and gender-specific BMI-z scores were calculated based on the least mean square method (LMS) (25, 26). Body fat percentage (BF%) and lean mass were estimated using the same body composition analyzer, which integrates a validated bio-impedance analysis method developed for an age range of 5–99 years old (27). The individual measurements were automatically converted to z-scores using the formula incorporated in the instrument (27). Three international cut-offs were applied to classify the weight status. The BMI classification was based on the World Health Organization (WHO) growth reference (25) and the updated International Obesity Task Force (IOTF) cut-off (26). The body fat reference established for segmental BIA by the Child Growth Foundation (CGF) was used to classify the adolescents' adiposity (28). The following cut-offs are used to define obesity, according to each reference: the z-score ≥+2 standard deviations by the WHO (25); the percentile curve passing through the BMI = 30 kg/m^2^ at age 18 by the IOTF (26), and the BF% ≥ 95th percentile by the CGF (28).

### Physical activity and sedentary behavior

Self-reported physical activity and sedentary behavior were assessed using a validated Malay version of the physical activity questionnaire for older children (PAQ-C), known as PAQ-C (M) (29). The questionnaire was adapted (30), modified, and validated among the 10–17 years old Malaysian students, with an acceptable validity of Spearman's correlation coefficient *r* = 0.6 (29). The subjects were required to complete questions related to their activities during leisure time, physical education (PE) classes, recess, lunchtime, after school, evenings, and weekends. They were also asked to provide a self-perception of their physical activity over the course of the seven days, with the final item asking the frequency with which they had participated in daily physical activity in the previous week. A five-point Likert scale ranging from low (score = 1) to high (score = 5) was used for each item. The physical activity score (PAQ score) is the average total score from these nine questions. A higher score indicates a greater physical activity level. Based on the PAQ score, each subject was categorized as having either low (PAQ score ranging from 1.00–2.33), moderate (PAQ score 2.34–3.66), or high (PAQ score 3.67–5.00) physical activity levels (31). For the current analysis, those with moderate or high physical activity levels were regarded as active, while adolescents with low physical activity were deemed inactive.

Regarding sedentary behavior, the adolescents were asked to report their daily sedentary activities (sitting or lying down) over the previous week, which included the number of hours usually spent per day during school days and on the weekend watching television, playing video games, surfing the internet/using the computer, doing homework/revision, attending extra classes (not within regular school hours), reading, and sitting while playing. Then, the average duration of total sedentary time was calculated for every subject. Due to the insufficient evidence available to specify time limits on sitting in children and adolescents, the recommended sitting time of not more than eight hours per day for adults was applied to classify the duration of sedentary behavior (32).

### Other lifestyle variables

The intake of specific types of dietary supplements—including fatty acids, growth/protein formula, and antioxidant-rich supplements—was extracted using a dietary supplement questionnaire (DiSQ) with a substantial kappa correlation coefficient (*k* = 0.62) (33). In brief, the questionnaire inquires about the consumption of vitamins/minerals and nutraceutical or natural products over the past year (using yes/no questions). The adolescents were then required to provide the product names. Sociodemographic information such as gender, date of birth, ethnic group, and type of school, as well as smoking and alcohol intake status, was obtained *via* the same questionnaire. Regarding antioxidant supplements, those known to have antioxidant properties are vitamin C, vitamin E, multi-vitamin, B-carotene, selenium, zinc, phenols, ubiquinone/co-enzyme Q10, and Ginkgo Biloba (34, 35). For the current analysis, the adolescents were identified as either occasional drinkers (defined as drinking at most once a month) or non-drinkers (defined as never, or tried once or twice). Meanwhile, smoking status was categorized as either regular smokers (at least one puff a day) or non-smokers (tried once, sometimes, or never) (23).

### Statistical analysis

At the initial stage, all the variables were tested for non-normality based on histograms and skewness values larger than two or an absolute kurtosis larger than seven (36). Continuous variables were presented using means and confidence intervals (CI), while categorical variables were presented using frequency statistics (the count and percentage). Gender-specific analysis was performed to assess the differences between the sexes. Analysis of covariance (ANCOVA) was conducted after taking into account age as a potential confounding factor. Meanwhile, an independent *t*-test was applied for age. Differences in proportions (percentages) were carried out using the *χ*^2^ test.

The strength of agreement/degree of concordance between each pair of cut-offs was calculated using the kappa (k) coefficient. Cohen's k coefficient values of less than 0.20 are described as poor agreement, values of 0.21–0.40 as fair agreement, values of 0.41–0.60 as moderate agreement, values of 0.61–0.80 as substantial agreement, and values of 0.81–1.00 as almost perfect agreement (37).

A multivariate linear regression model was generated to determine the best combination of lifestyle predictors for body fat percentage and lean mass, while a multivariate logistic regression model was created to determine the risk of obesity after controlling for confounding factors. Potential confounders were predetermined *via* the univariate analysis. Only significant variables in the univariate analysis were entered into the multivariate model. The variance inflation factor (VIF) was calculated for each regression model and detected no severe multicollinearity issues (>10) that warranted corrective measures.

Statistical analyses were performed using the SPSS for Windows version 24.0 (IBM Corp. Armonk, NY). A *p*-value of <0.05 is considered significant.

## Results

Table 1 shows the distribution of the subjects by gender and overall (*n* = 923). A total of 49.5% (457) participants were female adolescents, while 50.5% (466) were males. The overall composition was 65.4% Malays, 20.6% Chinese, 9.5% Indians, and 4.4% Orang Asli of the Semai tribe (the most prominent tribal group in Peninsular Malaysia). More than half of the sample population reported low levels of physical activity (60%), with 64% being identified as sedentary. In regard to gender, male and female adolescents did not differ by ethnicity, school type, sedentary level, or any type of dietary supplement intake. Female adolescents were less active than males (*p* < 0.05). Male adolescents reported higher proportions of smoking and alcohol consumption compared to female adolescents (*p* < 0.05). Male adolescents had higher mean lean mass and PAQ scores (*p* < 0.005), while female adolescents had a higher mean fat percentage than male adolescents (*p* < 0.05). There were no significant differences between the genders regarding the mean age, BMI-z scores, and sedentary time.

**Table 1:** Sample population characteristics (*n* = 923).

	Female (*n* = 457)	Male (*n* = 466)	All (*n* = 923)
	Mean (±CI) or *N* (%)	Mean (±CI) or *N* (%)	Mean (±CI) or *N* (%)
Age (year)^a^	15.93 (15.88–15.98)	15.98 (15.92–16.03)	15.95 (15.92–15.99)
Ethnicity^b^			
Malay	312 (68.2)	292 (62.7)	604 (65.4)
Chinese	83 (18.2)	106 (23.2)	191 (20.7)
Indian	37 (8.1)	50 (10.7)	87 (9.5)
Orang Asli	25 (5.5)	16 (3.4)	41 (4.4)
School^b^			
Boarding	63 (13.8)	73 (15.7)	136 (14.7)
Daily-urban	199 (43.5)	191 (41.0)	390 (42.3)
Daily-rural	153 (33.5)	143 (30.7)	296 (32.1)
Sport	42 (9.2)	59 (12.7)	101 (10.9)
Height (cm)^c^	155.71 (155.16–156.27)*	166.05 (165.43–166.68)	160.93 (160.40–161.47)
BMI-z score^c^	0.14 (0.01–0.27)	−0.01 (−0.14–0.12)	0.06 (−0.03–0.16)
Fat percentage (%)^c^	28.37 (27.669–29.08)*	13.20 (12.50–13.89)	20.71 (20.02–21.41)
Lean mass (kg)^c^	37.38 (36.78–37.99)*	50.44 (49.84–51.03)	43.97 (43.37–44.57)
PAQ score^c^	2.04 (1.98–2.09)*	2.38 (2.32–2.43)	2.21 (2.17–2.24)
Physical activity level^b^			
Less active	339 (74.2)*	211 (45.3)	550 (59.6)
Active	118 (25.8)	255 (54.7)	373 (40.4)
Sedentary time (hour/day)^c^	8.64 (8.48–8.81)	8.41 (8.25–8.58)	8.53 (8.41–8.64)
Sedentary behavior^b^			
Low	160 (35.0)	177 (38.0)	337 (36.5)
High	297 (65.0)	289 (62.0)	586 (63.5)
DS-Antioxidant^b^			
Yes	85 (18.6)	68 (14.6)	153 (16.6)
No	372 (81.4)	398 (85.4)	770 (83.4)
DS-Growth formula^b^			
Yes	8 (1.8)	17 (3.6)	25 (2.7)
No	449 (98.2)	449 (96.4)	898 (97.3)
DS-Fatty acids^b^			
Yes	27 (5.9)	19 (4.1)	46 (5.0)
No	430 (94.1)	447 (95.9)	877 (95.0)
Smoking status^b^			
Yes (regular)	0 (0.0)*	49 (10.5)	49 (5.3)
No	457 (100.0)	417 (89.5)	874 (94.7)
Alcohol status^b^			
Yes (occasional)	23 (5.0)*	50 (10.7)	73 (7.9)
No	434 (95.0)	416 (89.3)	850 (92.1)

^a^ Independent *t*-test.

^b^ *χ*^2^ test.

^c^ ANCOVA, Analysis of covariance, adjusted for age.

* Significant differences between genders (*p* < 0.05). BMI-z, Age- and gender-specific Body mass index; PAQ, Physical activity questionnaire; DS, Dietary supplement; CI, confidence interval.

Table 2 displays the prevalence of nutritional status based on the three international references. The prevalence of overweight/obese was 24.3% for the WHO, 22.5% for the IOTF, and 26.3% for the CGF. Meanwhile, the prevalence of normal weight was 69.6% for the WHO, 61.8% for the IOTF, and 51.9% for the CGF. No statistical differences were identified between the genders except for the CGF classification, where male adolescents had a greater prevalence of underfat (41%) than females (2%) (*p* < 0.05).

**Table 2:** Prevalence of nutritional status [N (%)] based on the three international references (*n* = 923).

	BMI-WHO			BMI-IOTF			CGF		
	Male	Female	All	Male	Female	All	Male	Female	All
Underweight/fat	37 (7.9)	20 (4.4)	57 (6.2)	79 (17.0)	66 (14.4)	145 (15.7)	191 (41.0)	10 (2.2)*	201 (21.8)
Normal	319 (68.5)	323 (70.7)	642 (69.6)	289 (62.0)	281 (61.5)	570 (61.8)	196 (42.1)	283 (61.9)	479 (51.9)
Overweight/fat	59 (12.7)	66 (14.4)	125 (13.5)	58 (12.4)	69 (15.1)	127 (13.8)	31 (6.7)	72 (15.8)	103 (11.2)
Obese	51 (10.9)	48 (10.5)	99 (10.7)	40 (8.6)	41 (9.0)	81 (8.8)	48 (10.3)	92 (20.1)	140 (15.2)

* Significant differences between genders (*p* < 0.05) using *χ*^2^. BMI, Body mass index; WHO, World Health Organization; IOTF, International Obesity Task Force; CGF, Child Growth Foundation.

Table 3 exhibits a comparison of the overweight/obese prevalence between the three international references. The agreement between WHO-IOTF was very good (*k* = 0.95), while the agreement between WHO-CGF (*k* = 0.76) and IOTF-CGF (*k* = 0.77) was good. A good to very good agreement between the three references allowed any subsequent analysis to employ only one reference.

**Table 3:** Comparison between the international references.

Reference comparison	Percent Agreement (%)	Kappa (k) coefficient
WHO-IOTF	98.3	0.95
WHO-CGF	91.0	0.76
IOTF-CGF	91.4	0.77

WHO, World Health Organization; IOTF, International Obesity Task Force; CGF, Child Growth Foundation.

Table 4 demonstrates the univariate and multivariate linear regression models for body fat percentage. After adjusting for significant confounders (gender, race, and school type), no lifestyle determinant was found to be a significant predictor of fat percentage (*R*^2^ = 0.506, *p* < 0.001).

**Table 4:** Linear regression analyses for factors associated with body fat percentage (*n* = 923).

	Univariate			Multivariate adjusted		
	B	95% CI	*p*-value	B	95% CI	*p*-value
Age (year)	−0.10	−1.28–1.08	0.868	–	–	–
Gender						
Female	Ref			Ref		
Male	−15.16	−16.15– −14.17	<0.001	−14.97	−16.04– −13.91	<0.001
Ethnicity						
Malay	Ref		0.033	Ref		
Chinese	−2.19	−3.937– −0.44	0.014	−1.38	−2.89–0.14	0.075
Indian	−0.71	−3.128–1.71	0.563	1.21	−0.73–3.15	0.223
Orang Asli	2.29	−1.109–5.69	0.186	1.69	−0.89–4.28	0.199
School						
Daily-urban	Ref		0.031	Ref		
Boarding	1.13	−0.97–3.23	0.291	1.38	−0.24–3.01	0.096
Sport	−2.95	−5.30– −0.60	0.014	−1.87	−3.80–0.07	0.058
Daily-rural	−0.01	−1.64–1.61	0.990	−1.10	−2.49–0.29	0.122
PAQ score	−4.06	−5.21– −2.91	<0.001	−0.10	−1.05–0.84	0.830
Sedentary time (hour/day)	0.25	−0.14–0.63	0.206	–	–	–
Smoking status						
No	Ref			Ref		
Yes (regular)	−8.75	−11.81– −5.70	<0.001	−0.97	−3.28–1.34	0.410
Alcohol Status						
No	Ref			Ref		
Yes (occasional)	−2.84	−5.41– −0.27	0.031	0.59	−1.44–2.62	0.571
DS-Antioxidant						
No	Ref			–	–	–
Yes	−0.01	−1.88–1.86	0.992	–	–	–
DS-Growth formula						
No	Ref			–	–	–
Yes	−2.96	−7.24–1.33	0.176	–	–	–
DS-Fatty acids						
No	Ref			–	–	–
Yes	−0.30	−3.50–2.90	0.854	–	–	–
R^2^	** **	** **	** **	** **	0.506	<0.001

CI, Confidence interval; B, Adjusted unstandardized regression coefficient; R^2^, Model’s coefficient of multiple determination; PAQ, Physical activity questionnaire; DS, Dietary supplement; Ref, Reference.

Table 5 shows the univariate and multivariate linear regression models for lean mass. After adjusting for selected demographic factors (gender, race, and school type), only one lifestyle indices, namely the physical activity scores, was found to be significant as a positive predictor of lean mass (*p* = 0.027). This model accounted for 53% of the variation in the adolescent lean mass (*R*^2^ = 0.531, *p* < 0.001).

**Table 5:** Linear regression analyses for factors associated with lean mass (*n* = 923).

	Univariate			Multivariate adjusted		
	B	95% CI	*p*-value	B	95% CI	*p*-value
Age (year)	1.49	0.48–2.50	0.004	0.83	0.03–1.62	0.041
Gender						
Female	Ref			Ref		
Male	13.10	12.25–13.95	<0.001	12.58	11.68–13.47	<0.001
Ethnicity						
Malay	Ref		<0.001	Ref		
Chinese	1.32	−0.17–2.80	0.082	0.09	−1.18–1.37	0.885
Indian	−0.52	−2.57–1.53	0.619	−1.20	−2.83–0.44	0.151
Orang Asli	−8.42	−11.31– −5.54	<0.001	−5.87	−8.04– −3.69	<0.001
School						
Daily-urban	Ref		<0.001	Ref		
Boarding	1.41	−0.38–3.20	0.123	0.16	−1.34–1.66	0.836
Sport	2.10	0.87–4.10	0.041	−0.15	−1.84–1.54	0.860
Daily-rural	−2.30	−3.69– −0.91	0.001	−1.50	−2.70– −0.31	0.014
PAQ score	3.98	3.00–4.97	<0.001	0.87	0.08–1.66	0.032
Sedentary time (hour/day)	−0.05	−0.38–0.28	0.752	–	–	–
Smoking status						
No	Ref			Ref		
Yes (regular)	6.85	4.21–9.49	<0.001	−0.03	−1.97–1.91	0.977
Alcohol Status						
No	Ref			Ref		
Yes (occasional)	2.82	0.61–5.04	0.013	0.70	−0.99–2.40	0.416
DS-Antioxidant						
No	Ref			–	–	–
Yes	−1.20	−2.81–0.41	0.145	–	–	–
DS-Growth formula						
No	Ref			Ref		
Yes	4.27	0.58–7.95	0.023	1.06	−1.54–3.67	0.423
DS-Fatty acids						
No	Ref			–	–	–
Yes	−1.65	−4.40–1.11	0.241	–	–	–
R^2^					0.531	<0.001

B, Adjusted unstandardized regression coefficient; CI, Confidence interval; R^2^, Model’s coefficient of multiple determination; PAQ, Physical activity questionnaire; DS, Dietary supplement; Ref, Reference.

Table 6 displays the univariate and multivariate logistic regression models for obesity risk. After adjusting for a significant demographic confounder (school type), adolescents who sat for more than eight hours a day had obesity odds 2.3 times higher than those who sat for less than eight hours a day (*p* = 0.003), while adolescents who took antioxidant-type supplements had obesity odds 0.4 times lower than their non-intake counterparts (*p* = 0.010).

**Table 6:** Logistic regression analyses for factors associated with the risk of obesity^a^ (*n* = 923).

Characteristics	Univariate			Adjusted-multivariate		
	OR	95% CI	*p*-value	OR	95% CI	*p*-value
Age (year)	0.72	0.50–1.02	0.065	–	–	–
Gender						
Female	1.0	Ref		–	–	–
Male	1.05	0.70–1.59	0.829	–	–	–
Ethnicity						
Malay	1.0	Ref		–	–	–
Chinese	0.66	0.38–1.17	0.159	–	–	–
Indian	0.95	0.47–1.91	0.874	–	–	–
Orang Asli	0.00	0.00–0.00	0.998	–	–	–
School						
Daily-urban	1.0	Ref		1.0	Ref	
Boarding	1.03	0.59–1.82	0.911	1.34	0.74–2.43	0.328
Sport	0.26	0.09–0.74	0.012	0.40	0.13–1.21	0.106
Daily-rural	0.54	0.32–0.90	0.017	0.49	0.29–0.82	0.007
Physical activity level						
Active	1.0	Ref		–	–	–
Less-active	1.56	1.00–2.43	0.052	–	–	–
Sedentary behavior						
Low	1.0	Ref		1.0	Ref	
High	2.31	1.40–3.82	<0.001	2.27	1.32–3.93	0.003
Smoking status						
No	1.0	Ref		–	–	–
Yes (regular)	0.73	0.26–2.07	0.553	–	–	–
Alcohol status						
No	1.0	Ref		–	–	–
Yes (occasional)	1.03	0.48–2.21	0.947	–	–	–
DS-Antioxidant						
No	1.0	Ref		1.0	Ref	
Yes	0.47	0.23–0.96	0.038	0.39	0.19–0.80	0.010
DS-Growth formula						
No	1.0	Ref		–	–	–
Yes	0.718	0.17–3.09	0.657	–	–	–
DS-Fatty acids						
No	1.0	Ref		–	–	–
Yes	0.18	0.02–1.30	0.088	–	–	–

^a^ WHO, World Health Organization cut-off; OR, Odds ratios; CI, Confidence interval; DS, Dietary supplement; Ref, Reference.

## Discussion

The present study estimated a high prevalence of overweight and obesity in adolescents aged 15–17. The prevalence of obesity in a national study among children and adolescents under 18 (15%) was slightly higher than that reported in this study (11%), probably due to the wider age range (5, 38).

Notably, a higher prevalence of overweight/obesity was discovered with the CGF cut-off (26%), followed by those of the WHO (24%) and IOTF (23%). A previous comparison study also found important differences between the cut-off points for BMI and body fat. The prevalence of overweight/obesity in children and adolescents aged 7–19 from Southern Europe (Italy and Spain) was 32.3% according to IOTF, 37.3% according to the WHO, and 39.8% according to the CGF references (39). The discrepancies suggest the importance of specifying the method and reference cut-off points used to classify a sample until a consensus is reached. Despite the differences, the concordance values between each pair were high. Currently, there is no recommended/standard cut-off for the evaluation of overweight or obesity. Therefore, the high concordance values allowed for the application of any cut-off.

Concerning the risk of obesity, the results from the multivariate analysis revealed elevated odds of obesity when sedentary levels were high. Sitting for more than eight hours per day increased the odds of obesity by 2.3 times. The findings of the current study corroborated those reported among adolescents in France (40), the USA (41), Southeast Asia (42, 43), and Lebanon (44, 45). Other studies also demonstrated a link between long screen time and obesity (44, 46). However, several studies have also reported no significant association between obesity and sedentary behavior/ long screen-time in adolescents (47–49). The reason for this contradictory result is still not entirely apparent but could be partly attributed to methodological differences. Sedentary behavior involves very low energy expenditure and severely limits the number of calories burnt. Prolonged sitting could suppress lipoprotein lipase (a protein important for controlling plasma triglyceride catabolism, HDL cholesterol, and other metabolic risk factors) activity due to the loss of contractile stimulation of weight-bearing muscle, which could contribute to the development of obesity in the long term (50, 51). Whether exercise can reverse the detrimental effects of prolonged sitting requires more high-quality control trial studies. However, the current recommendation is to interrupt long hours of sitting with light movements (52).

The protective role of physical activity against obesity may not be consistent among adolescents. Interestingly, the analysis of the components of body composition revealed that physical activity contributes significantly to an increase in lean mass. Muscle growth or muscle hypertrophy is linked to a physiological process known as muscle protein synthesis, in which protein is produced to repair the muscle damage caused by intense exercise (53). This recovery process occurs after exercise, when the body is at rest. Muscle hypertrophy occurs whenever the rate of muscle protein synthesis is greater than the rate of muscle protein breakdown (54). Thus, the impact of exercise when repeated over time eventually contributes to muscle hypertrophy (53, 54).

When comparing weight-loss intervention programs, which are widely accepted methods for dealing with obesity, physical activity interventions alone appear to be less effective than combined intervention programs that include a caloric-restriction/hypocaloric (diet) and exercise (either resistance or endurance) (55). Although diet-only interventions and combined intervention programs produced similarly increased weight loss (55–57), diet-induced weight loss alone may significantly decrease muscle mass (58, 59). On the other hand, diet and physical activity combined programs were found to be not only effective in reducing fat mass but also able to attenuate muscle loss (56–58). Moreover, physical activity, particularly resistance exercise, not only protects muscle tissue during weight loss but may also increase muscle strength (56, 59, 60). This information is important since compared to a normal-weight person, those who are obese have higher muscle mass but lower muscle quality. Maximum muscle hypertrophy can be achieved through a combination of physical activities (such as resistance training) and hyperaminoacidemia from protein intake (54). However, protein intake alone cannot increase muscle strength. Accordingly, participation in physical activity (or in conjunction with protein intake) is more advisable than simply taking protein supplements to increase muscle mass.

Of the three supplement categories explored, antioxidant supplements were found to protect against obesity. Research indicates that low circulatory concentrations of antioxidants in the blood are linked with increased body fat and BMI in children (61). Meanwhile, others have highlighted that overweight and obese individuals have lower concentrations of antioxidants in their blood than children and adolescents of normal weight (62–64). Excess body fat may enhance oxidative stress, which depletes endogenous and exogenous antioxidants *via* the increased indirect utilization of antioxidants, while the free radicals generated may play a major role in the etiology and development of obesity-related comorbidities. However, the proposed mechanism should consider the limitations of the existing studies (65). Based on the assumption that individuals with excess body fat have high oxidative stress, overweight/fat and obese individuals with low antioxidant levels in the body to act as direct antioxidants are advised to consume more antioxidants (as food, or supplements intake, or a combination of these) to attain the same serum-plasma levels as a normal-weight person (66). These observational studies in children demonstrate that antioxidants may have an impact on obesity, but causality can only be ascertained with clinical trials.

Several limitations of the present study should be acknowledged. Firstly, dietary patterns were not investigated. Other local studies have reported that a dietary pattern mainly characterized by high sugar and fat among adolescents was not associated with obesity (67, 68). The present study found no definitive association between physical activity and obesity. Similar findings have been reported by other studies (40, 41, 43, 44, 46, 47, 49). Meanwhile, some studies have noted a significant association between physical activity and obesity (17, 18, 19, 45, 48, 69). These discrepancies could be related to recall bias from self-reporting methods (although validated), hence the use of an objective measure is recommended, although this is impractical for large-scale epidemiological studies. Moreover, the PAQ-C is limited to measuring the level of physical activity and cannot discriminate between types of physical activity (e.g., endurance vs. resistance exercise). Additionally, no causal inferences could be made as the data were drawn from a cross-sectional study. Longitudinal studies are warranted to confirm any actual links. Despite these limitations, the present study not only represents multi-ethnic adolescents in Malaysia but also, most importantly, assessed the odds of being obese and other body composition parameters, including lean mass and fat percentage, which had not been reported previously. In the wake of the new norms, Malaysia is at high risk of widespread inactivity and an obesity pandemic. Moreover, many experts contend that early healthy lifestyle practices tend to be translated into adulthood and indirectly reduce the burden of disease throughout life (70, 71). Considering that, the current findings indicate that urgent action is needed, with extensive focus on strategies that can help adolescents to create and follow healthy lifestyles.

## Conclusion

The current study demonstrated a high prevalence of overweight and obesity among Malaysian adolescents aged 15–17. Although the prevalence of obesity differs based on the reference method employed, the degree of concordance between each pair was high. Excessive sedentary behavior was found to increase the odds of obesity, while antioxidant-rich supplements were found to protect against obesity. A high lean mass was positively associated with physical activity (PAQ score), while no lifestyle determinant was found to be a significant predictor of fat percentage. The lifestyle predictors identified in this study could be considered when designing interventions that integrate lifestyle modifications targeting adolescents.

## Data Availability

The original contributions presented in the study are included in the article/Supplementary Material, further inquiries can be directed to the corresponding author/s.
